# Creep Damage Characteristics of Fiber-Reinforced Alkali-Activated Slag Concrete: Effect of Age and Stress

**DOI:** 10.3390/ma19040722

**Published:** 2026-02-13

**Authors:** Ziyang Zhang, Sikai Wu, Xianggang Bian, Jianfei Kang, Jianbo Guo

**Affiliations:** 1 School of Civil Engineering, Suzhou University of Science and Technology, Suzhou 215009, China; 2 State Key Laboratory of Safety, Durability and Healthy Operation of Long Span Bridges, Southeast University, Nanjing 210096, China; 3 School of Civil Engineering, Tongji University, Shanghai 200092, China; 4 School of Civil Engineering, Southwest Jiaotong University, Chengdu 610031, China

**Keywords:** compressive creep, creep damage, nonlinear creep, fiber reinforced alkali activated slag concrete

## Abstract

This study investigates the effects of fiber reinforcement, stress levels, and curing age on the creep behavior of alkali-activated slag (AAS) concrete. Through comprehensive cyclic loading tests, we demonstrate that fiber reinforcement significantly reduces irreversible creep strain by 1.2–5.3% under high-stress conditions (0.7$f_{c}$), with optimal performance at 1.0% fiber content. Quantitative analysis reveals that fiber-reinforced specimens exhibit 10.0% higher elastic modulus and maintain 83% of peak strength after creep damage, compared to 86% strength retention in non-fiber specimens. Ultrasonic testing confirmed that fibers effectively mitigate internal damage under high stress, limiting wave propagation time increases to 47–62% versus 66% in plain AAS concrete. This research quantifies the pronounced age sensitivity of creep behavior, with 7-day specimens exhibiting 28% higher creep strain than 28-day specimens under 0.8$f_{c}$ stress, corresponding to irreversible strain ratios of 21.3% and 18.4%, respectively. A 102% increase in Poisson’s ratio at high stress levels provides direct evidence of fiber-controlled volumetric expansion during microcracking. These findings establish that strategic fiber incorporation fundamentally alters the creep damage mechanisms in AAS concrete, providing critical quantitative thresholds for engineering applications subjected to sustained loading. The results offer practical guidance for optimizing fiber-reinforced AAS concrete in infrastructure requiring long-term dimensional stability.

## 1. Introduction

The cement industry contributes approximately 7.4% of global CO_2_ emissions [1], primarily from clinker production [2]. To mitigate environmental impacts, geopolymer concrete (GPC), synthesized from industrial by-products such as fly ash and silica fume, has been proposed as a sustainable alternative [3]. However, the absence of standardized design codes currently restricts its application in structural components [4].

Creep behavior remains a critical factor in determining the performance and durability of concrete structures. Creep and shrinkage substantially influence stiffness and service life, and insufficient design considerations can result in cracking, load reduction, and premature deterioration [5,6,7]. Studies on GPC creep have yielded inconsistent results. Wallah and Hardjito reported creep coefficients of 0.3–0.5 for heat-cured specimens, indicating lower creep than ordinary Portland concrete (OPC) [8,9]. In contrast, Gunasekera and Zhang observed similar creep behavior in both binders [10,11]. Micromechanical investigations provide further insight: Wallah attributed creep to unreacted fly ash particles [8], Lee identified sodium aluminosilicate hydrate phases as highly compliant [12], and Chen linked creep magnitude to nanoscale pore distributions influenced by activator modulus [13]. These findings highlight the sensitivity of GPC creep to material composition, curing conditions, and microstructural characteristics.

The incorporation of fibers has been widely studied as a means of mitigating creep deformation in concrete systems. Polypropylene fibers are particularly attractive because of their low dosage requirements and reduced cost, providing an economical reinforcement option [14,15]. Mechanistically, fibers constrain matrix deformation through interfacial adhesion, with their effectiveness strongly influenced by the applied stress-strength ratio [16]. Recent studies confirm that steel fibers (1.0% dosage) reduce 28-day creep strain by 32% in alkali-activated slag (AAS) concrete via crack-bridging and interfacial adhesion mechanisms [17], while advanced manufacturing techniques, such as optimized 3D printing parameters (e.g., 1.5 mm layer thickness, 35 °C nozzle temperature), further enhance creep resistance by improving interlayer bonding and reducing long-term strain by 22% [18]. Beyond compressive creep, tensile creep studies reveal that sustained loading induces progressive interfacial degradation between the matrix and aggregates in AAS systems, significantly contributing to time-dependent deformation [19]. Geometric factors also modulate creep response, with increasing volume-to-surface ratio (1.5 to 3.0 m^−1^) reducing creep strain by 37%, effectively captured by the nominal size parameter in predictive models [20].

Creep not only causes time-dependent deformation but also induces progressive damage that may trigger structural failure. Rusch [21] observed that sustained loading reduces strength, while Smadi et al. [22,23] demonstrated that failure occurs once critical strain thresholds, closely aligned with peak short-term strains, are exceeded. Mazzotti and Savoia [24] further confirmed accelerated creep development under high stress ratios, and Neville et al. [25] classified creep into three stages: decelerating, steady-state, and accelerated, the latter preceding collapse. Systematic investigations quantified that creep strain in AAS systems increases approximately 45% when the stress-strength ratio elevates from 0.4 to 0.7, with the MC2010 model showing strong predictive capability (deviation < 5%) by accounting for time-dependent microstructural evolution [20,26]. Despite extensive research, consensus on critical stress thresholds remains elusive. Reported values vary widely, from 40% to 90% [27,28], with intermediate thresholds proposed by Bazant [6], Rossi [29], and Iravani [30]. These discrepancies underscore the complexity of nonlinear creep and the need for context-specific assessment. Hussain’s research demonstrates that steel fibers effectively mitigate crack-induced deterioration in concrete by reducing water permeability and altering crack geometry, specifically by increasing surface roughness and decreasing tortuosity [31]. Given AAS concrete’s significantly lower carbon footprint and growing adoption in sustainable construction, comprehensive characterization of its creep performance under diverse service conditions, particularly the interplay of fiber reinforcement, sustained high stress, and geometric constraints, is imperative for reliable structural design. In this context, fiber-reinforced AAS systems show strong potential, as fibers redistribute stresses and regulate strain development. However, a critical knowledge gap persists regarding the quantitative relationships among fiber dosage, sustained stress magnitude, and curing age in polypropylene fiber-reinforced alkali-activated slag concrete, especially the lack of established correlations between macroscopic creep deformation and microstructural damage evolution under cyclic high-stress conditions.

This study systematically investigated the influence of fiber content, sustained stress levels, and specimen age on the creep behavior of polypropylene fiber-reinforced alkali-activated slag (FRAAS) concrete. Cyclic loading tests were conducted on specimens with three fiber dosages (0%, 0.5%, and 1.0% by volume), six stress-strength ratios (0.3, 0.4, 0.5, 0.6, 0.7, and 0.8), and three curing ages (7 d, 14 d, and 28 d) to examine parameter-dependent variations in stress-strain relationships. A systematic analysis of the test results revealed the effects of damage on the mechanical properties and micro structural characteristics.

## 2. Materials and Specimens

### 2.1. Materials and Mix Design

The AAS concrete used in this study was prepared with S105-grade ground granulated blast-furnace slag (GGBFS). The GGBFS used in this study was supplied by Shandong Changshengyuan Slag Micro-powder Co., Ltd. (Heze, China). The particle size distribution of the material, as determined by laser diffraction analysis, shows a $D_{10}$ of approximately 1.2 µm, $D_{50}$ of 12.5 µm, and $D_{90}$ of 48.3 µm. These characteristics comply with the requirements for GGBFS specified in GB/T 18046-2017 [32]. The chemical oxide composition of the GGBFS was analyzed using X-ray fluorescence (Bruker XRF spectrometer, Bruker, Billerica, MA, USA) spectroscopy. The slag powder contained 36.96% CaO, 27.42% SiO_2_, and 18.44% Al_2_O_3_. These oxide proportions satisfy the requirements for AAS systems [33]. The aggregate used in the tests was standard sand conforming to ISO 679 [34] specifications. The alkali activator comprised powdered sodium hydroxide (NaOH) and liquid sodium silicate solution. The NaOH powder was sourced from Sinopharm Chemical Reagent Co., Ltd. (Shanghai, China), with a purity exceeding 98.0% and chloride impurity content below 0.005%. The liquid sodium silicate was supplied by Foshan Kangning New Materials Technology Co., Ltd. (Foshan, China). A polycarboxylic acid-based polycarboxylate superplasticizer was used to improve workability. The fibers used were fine polypropylene (PP) monofilaments, each 12 mm in length. Key material properties of the GGBFS, sodium silicate solution, and PP fibers are presented in Table 1.

### 2.2. Mix Design and Specimen Preparation

The water-to-binder ratio of the AAS concrete mix was set at 0.40 to promote early strength development, requiring a higher slag content. All the water in the mix originated from the alkali activators, namely, NaOH solution and sodium silicate solution. The solid content of both the slag and activators was considered in the cementitious material mass calculations. This study investigated three fiber volume fractions (0%, 0.5%, and 1.0%). The mix proportions of the AAS concrete are presented in Table 2.

### 2.3. Specimen Preparation and Curing

**Figure 1:**
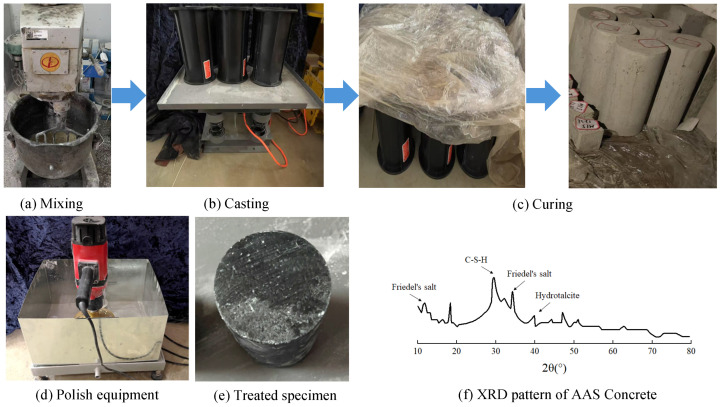
Specimen preparation process of alkali-activated slag concrete specimens [35].

The preparation of AAS concrete followed a dry-mixing procedure comprising three sequential steps, as shown in Figure 1a–c. First, all dry materials, including aggregates and ground granulated blast-furnace slag (GGBFS), were precisely weighed and placed into a concrete mixer for dry blending. Next, after achieving homogeneity in the dry mixture, polypropylene (PP) fibers were added, and the mixture was blended for an additional minute to ensure uniform fiber distribution. Finally, the alkali activator was gradually introduced into the mixture, followed by continuous mixing for three minutes to ensure uniform activation.

Upon completion of mixing, the fresh concrete was poured into pre-cleaned and lubricated molds. The mixture was compacted using a vibration table and finished with a trowel to attain a smooth surface. Immediately afterward, the specimens were sealed with plastic film and subjected to pre-curing in a controlled environment at 20 ± 1 °C and 95% relative humidity. After 24 h of pre-curing, the specimens were demolded, rewrapped with plastic film, and returned to the curing chamber until reaching the designated testing ages. Prior to testing, the top and bottom surfaces of each specimen were polished to minimize the impact of surface irregularities on test outcomes, as shown in Figure 1d,e. The XRD pattern of the AAS concrete shown in Figure 1f revealed the presence of C-S-H gel as the dominant hydration product, indicated by the broad hump around 29–30° 2θ. Minor crystalline phases identified include Friedel’s salt (peaks at 10° and 35° 2θ), associated with sulfate incorporation, and hydrotalcite (peak near 40° 2θ), suggesting the formation of magnesium-rich hydroxycarbonate phases under alkaline conditions.

## 3. Creep Damage Testing: Specimens and Loading Protocol

### 3.1. Creep Specimens

This study conducted a series of creep damage tests on AAS concrete, systematically evaluating the effects of three key factors: fiber content, sustained stress levels, and specimen age. Since the hydration reaction of AAS concrete is nearly complete by 28 d, resulting in minimal subsequent changes to its creep characteristics, three loading ages (7 d, 14 d, and 28 d) were selected. The nonlinear nature of creep strain and damage development in concrete is attributed to microcracking under loads exceeding the linear elastic range [36]. According to the findings by Wallah et al. [8], Zhang et al. [11], and Un et al. [37], the nonlinear creep threshold for AAS concrete is in the range of 0.3$f_{c}$–0.4$f_{c}$. Therefore, specimens were categorized into two groups: a control group subjected to stress-strength ratios of 0.3$f_{c}$ and 0.4$f_{c}$ (where $f_{c}$ denotes the compressive strength of AAS concrete), representing linear creep behavior without significant damage, and a damage group subjected to 0.5$f_{c}$–0.8$f_{c}$, corresponding to high-stress regimes that induce progressive creep damage. Six stress-strength ratios (0.3, 0.4, 0.5, 0.6, 0.7, and 0.8) in total were tested to thoroughly investigate creep damage behavior.

All specimens were identified using a standardized coding system, XXGYYWFZZ, where XX represents the initial loading age (7 d, 14 d, or 28 d), G denotes AAS concrete, YY represents the stress-strength ratio (expressed as a percentage of the compressive strength of AAS concrete), W stands for the group of the tested specimens (C and T are the controlled and damage tested group, respectively.), F represents fiber inclusion, and ZZ represents the fiber volume fraction (0.0%, 0.5%, or 1.0% by volume). For example, G0750F05 corresponds to a fiber-reinforced AAS concrete specimen loaded at 7 d with a stress-strength ratio of 0.50$f_{c}$, where ‘F05’ represents 0.5% fiber content. Each test condition included three replicate cylindrical specimens (labeled GXXYYFZZ-1, GXXYYFZZ-2, and GXXYYFZZ-3) for statistical reliability, resulting in a total of 54 specimens across all combinations of age, stress-strength ratio, and fiber content, as summarized in Table 3 and Table 4.

### 3.2. Testing Equipment and Loading Protocol

Both mechanical property testing and creep damage tests on the concrete specimens were conducted using a universal testing machine (Shanghai Sansi Zongheng Machinery Manufacturing Co., Ltd., Shanghai, China). Cylindrical specimens, with dimensions of 100 mm in diameter and 200 mm in height (ϕ100 mm × 200 mm), were used for all mechanical and creep tests. The loading protocol adhered to the specifications outlined in the Chinese standard GB/T 50081-2019 [38]. All specimens were subjected to a loading rate of 6280 N/s.

**Figure 2:**
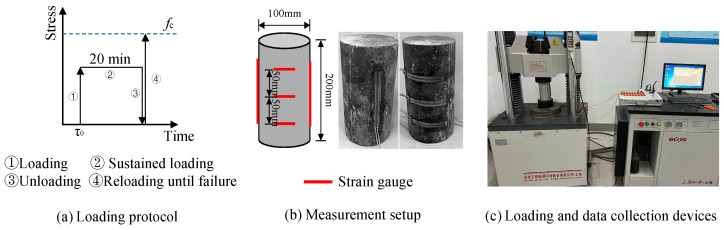
Creep loading protocol [35].

During the test, strain data were collected using strain gauges affixed to the specimen surfaces. Longitudinal strain was measured using 100 mm strain gauges symmetrically affixed to both sides of the specimens, while circumferential strain was captured by 80 mm strain gauges similarly positioned. The strain gauge data were continuously recorded using a data acquisition system throughout each test until specimen failure. The overall test setup is illustrated in Figure 2. The final results were derived from the average of three specimens to ensure statistical reliability.

### 3.3. Concrete Damage Detection Using Ultrasonic Method

**Figure 3:**
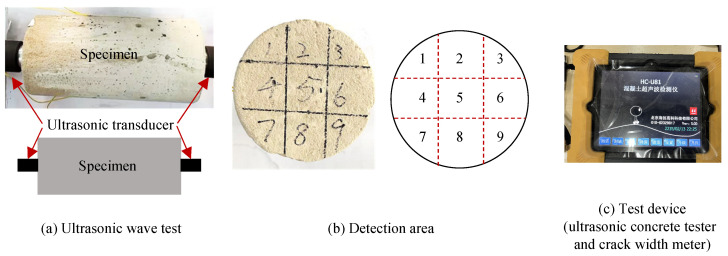
Using ultrasonic method for non-destructive testing of specimens [35].

For the creep-damaged specimens, non-destructive testing was conducted using an ultrasonic system after the sustained loading phase to assess crack development. As shown in Figure 3, the specimen surface was divided into nine regions to minimize the influence of random crack propagation. Ultrasonic transducers were positioned on each region to measure the propagation time of ultrasonic waves.

First, for the i-th region, baseline measurements were taken on intact specimens prior to testing to determine the initial propagation time $t_{0}^{i}$. Subsequently, measurements were repeated after unloading the specimens. The propagation time recorded during the *n*-th test was denoted as $t_{n}^{i}$. The average increase rate of propagation time $\bar{\Delta_{\tau }}$ was calculated as follows:

(1) $$\bar{\Delta \tau }=\frac{1}{9}\sum_{i=1}^{9}\frac{t_{n}^{i}-t_{0}^{i}}{t_{0}^{i}}$$

The increase in ultrasonic propagation time $\bar{\Delta \tau }$ indicates the extent of microcrack development within the concrete. This parameter offers a quantitative measure of internal damage accumulation caused by creep under sustained loading.

## 4. Results of the Experimental Program

### 4.1. Mechanical Properties

**Figure 4:**
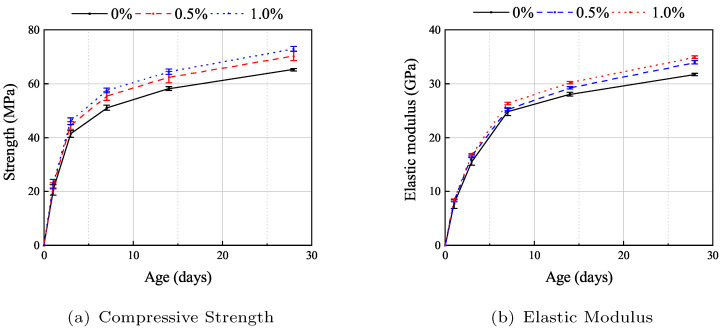
Development of the compressive strength and elastic modulus of the alkali-activated slag concrete [35].

Mechanical properties of AAS concrete were tested at 1 d, 3 d, 7 d, 14 d, and 28 d, with six specimens tested per age group (three for compressive strength and three for elastic modulus). Figure 4 illustrates the development of compressive strength and elastic modulus of AAS concrete with varying fiber volume fractions.

The results show that specimens with different fiber contents exhibit similar compressive strength evolution patterns, characterized by significant increases in both compressive strength and elastic modulus within the first 7 d, with elastic modulus development lagging slightly behind compressive strength. At 7 d, the compressive strength of specimens without fibers and with 0.5% and 1.0% fiber content reached 76%, 75%, and 78%, respectively, of their 28 d strengths. Similarly, their elastic modulus reached 72%, 75%, and 76% of the 28 d values. By 28 d, the compressive strengths were 65.20 MPa, 70.20 MPa, and 73.0 MPa for specimens without fibers and with 0.5% and 1.0% fiber content, respectively, while the corresponding elastic moduli were 31.7 GPa, 33.9 GPa, and 34.8 GPa.

The addition of fibers slightly enhanced both compressive strength and elastic modulus of AAS concrete. At 28 d, compared to the specimens without fiber, specimens with 0.5% fiber content achieved a maximum compressive strength increase of 7.7%. However, the rate of strength gain diminished with higher fiber content: adding 1.0% fiber increased compressive strength by only 3.9% compared to the 0.5% fiber group. In contrast, improvements in elastic modulus were more pronounced. At 28 d, elastic modulus increased by 6.9% and 10.0% for specimens with 0.5% and 1.0% fiber content, respectively, relative to the group without fiber. This is primarily attributed to the straight PP fibers, which are shorter than steel fibers and primarily bridge microcracks, delaying internal crack propagation under low stress, thus enhancing elastic modulus. However, under high stress, PP fibers (used in this study) exhibit lower tensile strength compared to other fibers (e.g., steel or basalt fibers), leading to weaker suppression of macrocracks and a minimal impact on compressive strength.

Strain gauges were attached to each specimen as it was monotonically loaded to failure to capture the stress–strain relationship during the loading process, thereby facilitating subsequent creep and damage analyses. Table 5 presents the peak strain, peak stress, and elastic modulus for each of these specimens. Note that the values in the table represent the averages of three specimens for each type.

### 4.2. Creep Processing

**Figure 5:**
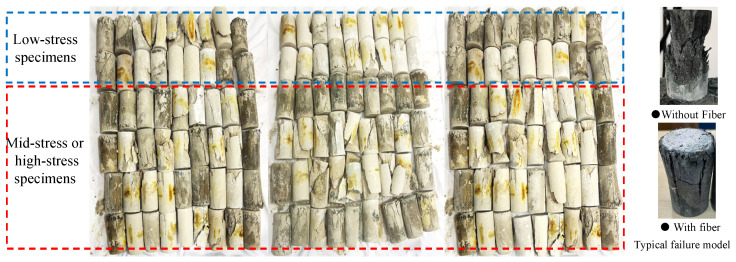
The specimens of the controlled group and the creep damage group.

As shown in Figure 5, creep damage tests were conducted on AAS concrete cylindrical specimens at 7 d, 14 d, and 28 d according to the testing protocol.

Due to variations in peak strain and stress among specimens of different ages, the stress-strain relationships were normalized using the average compressive strength and peak strain of each age group for comparative analysis. The normalization equations are defined as follows:

(2) $$\bar{\sigma }=\frac{\sigma_{c}}{\sigma_{0}},\bar{\varepsilon }=\frac{\varepsilon_{c}}{\varepsilon_{0}}$$

where $\bar{\sigma }$ and $\bar{\varepsilon }$ represent normalized stress and strain, $\sigma_{0}$ and $\varepsilon_{0}$ denote the peak stress and peak strain determined from monotonic loading tests, and $\sigma_{c}$ and $\varepsilon_{c}$ represent the stress and strain during creep testing.

**Figure 6:**
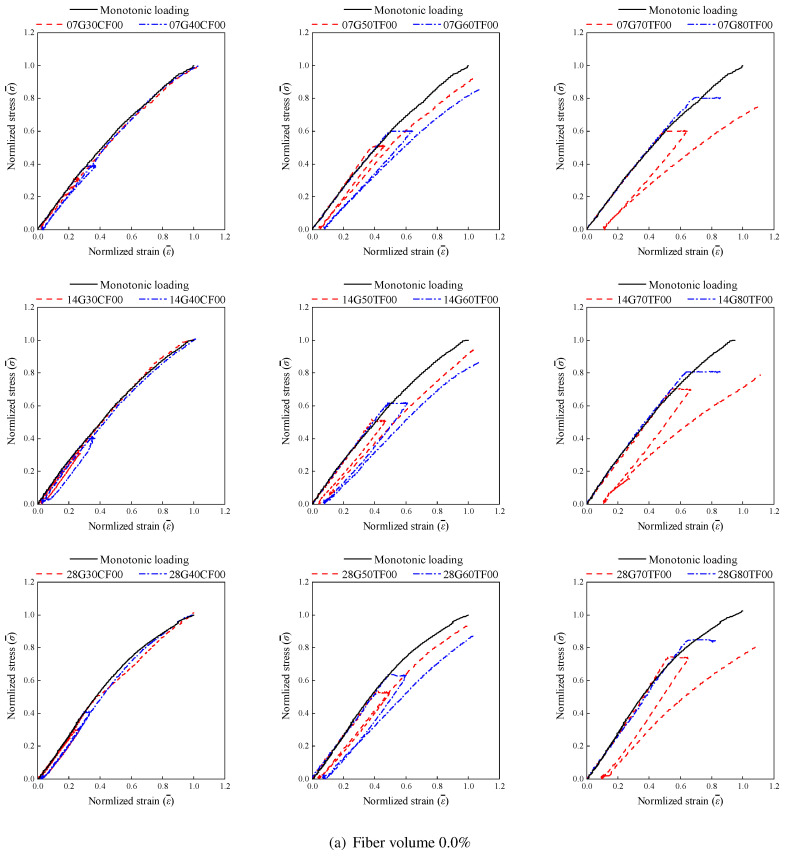

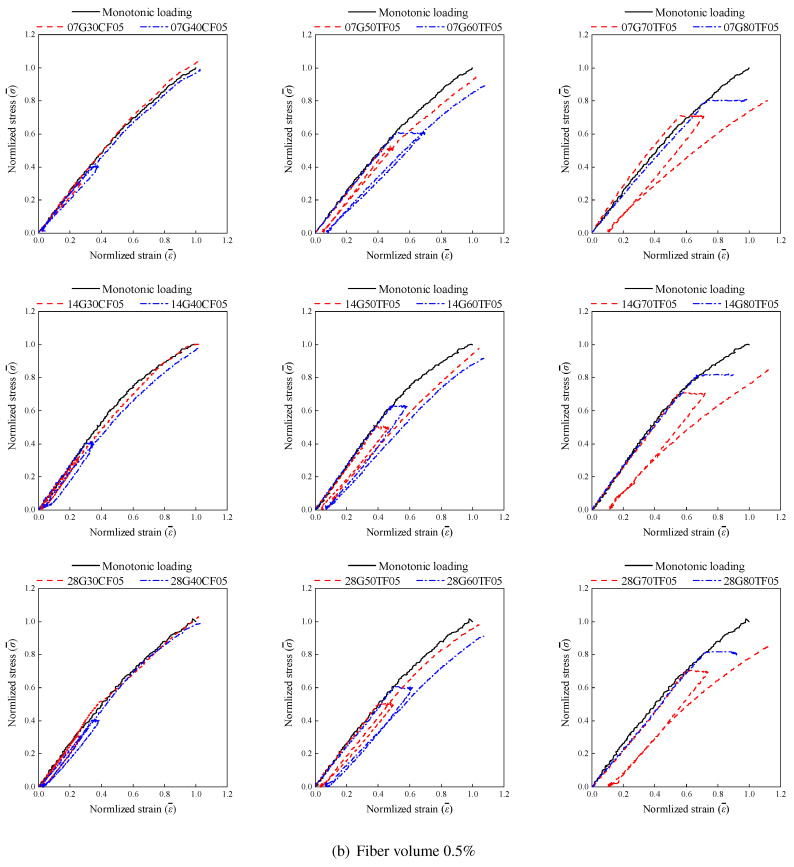

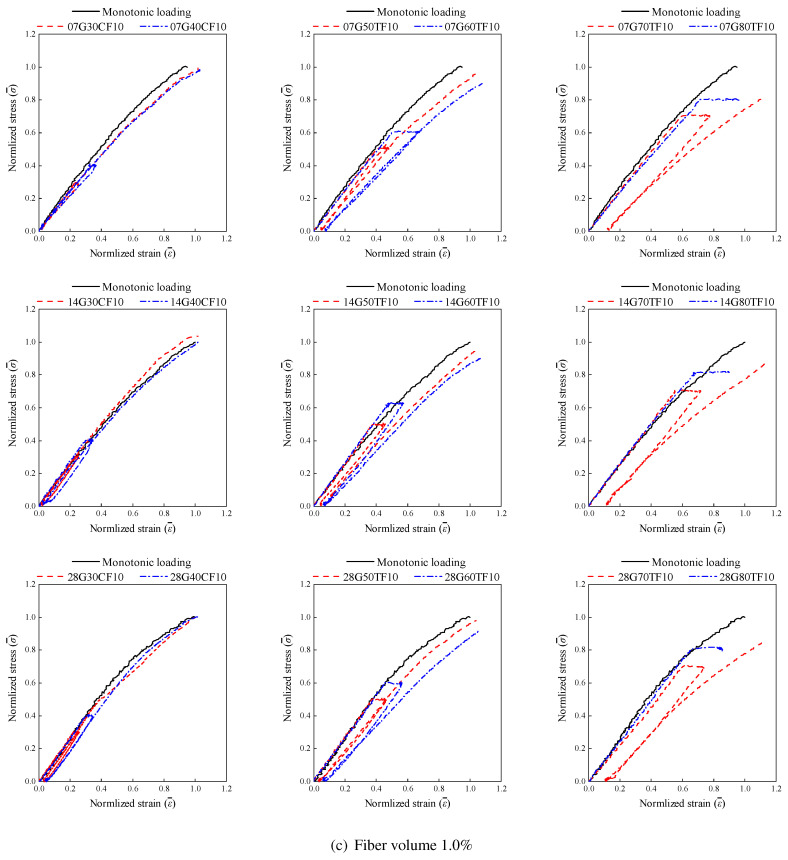
The normalized stress-strain curves of the tested specimens.

The normalized $\bar{\sigma }-\bar{\varepsilon }$ curves for control and creep-damaged specimens, shown in Figure 6, reveal distinct damage characteristics. In the initial loading phase, the curves of all specimens closely align with the monotonic loading curve, suggesting no creep damage. As sustained loading begins, mid-to-high stress specimens show internal creep damage. During unloading and reloading, deviations in the normalized curves become evident for some specimens, indicating significant damage accumulation. AAS concrete exhibits creep damage behavior similar to ordinary concrete: low-stress specimens maintain nearly parallel $\bar{\sigma }-\bar{\varepsilon }$ curves during loading and unloading, suggesting elastic deformation with minor irreversible creep strain. In contrast, mid-to-high stress specimens display increasing irreversible creep strain due to sustained loading. Notably, higher-stress specimens exhibit divergent loading and reloading curves, indicating severe damage, with microcracks propagating as stress levels increase. Furthermore, as the specimen age increases, the deviation between loading and unloading curves diminishes, reflecting reduced internal damage development rates over time.

To quantify creep damage progression, strain measurements were taken at three stages: initial strain $\varepsilon_{1}$ at the end of loading, final strain $\varepsilon_{2}$ after sustained loading, and irreversible strain $\varepsilon_{i}$ after unloading. The irreversible strain ratio $\varepsilon_{i}/\varepsilon_{1}$ was calculated and is shown in Table 6.

The control group under low stress exhibited minimal creep damage, with irreversible strain ratios ranging from 7.8% (28G30CF10) to 9.8% (07G40CF00). Mid-to-high stress specimens showed pronounced damage, with irreversible strain ratios ranging from 10.1% (28G50TF10) to 21.3% (07G70TF00). Specimen age significantly influenced $\varepsilon_{i}$: with increasing age, both irreversible strain ratios and their rates of change decreased. For 7 d, 14 d, and 28 d specimens, the maximum irreversible strain ratios under 0.7$f_{c}$ were 21.3% (07G70TF00), 21.0% (14G70TF00), and 18.4% (28G70TF00), respectively, demonstrating a declining trend.

As shown in Figure 6 and Table 6, fiber reinforcement had minimal impact on creep damage under low stress, with a maximum irreversible strain variation of 6.1%. Under elevated stress levels (0.7$f_{c}$), fibers reduced the irreversible strain ratios. Compared to specimens without fiber, adding 0.5% fibers reduced irreversible strain ratios by 2.4%, 3.8%, and 1.2% for 7 d, 14 d, and 28 d specimens, respectively. For 1.0% fiber content, reductions of 2.9%, 5.3%, and 2.8% were observed.

Microcrack development governs the observed creep damage patterns in AAS concrete. Under low-stress conditions, microcrack proliferation contributes to increased creep deformation despite minimal overall damage. In contrast, mid-to-high stress levels induce pronounced damage primarily through microcrack extension. Early-age high-stress loading inflicts substantial damage on AAS concrete via microcrack initiation and propagation mechanisms. The effectiveness of fiber reinforcement in mitigating creep damage under elevated stress aligns with mechanical property findings, confirming that fibers suppress crack propagation. This crack-inhibiting function becomes more significant with higher fiber content, particularly in younger specimens, demonstrating fibers’ critical role in enhancing the long-term durability of AAS concrete under sustained loading.

### 4.3. Creep Strain

Creep strain of specimens was measured during the sustained loading phase, and the creep coefficient at the end of loading was calculated, as summarized in Table 7.

As shown in Table 7, sustained loading induced measurable creep strain in all specimens even with a brief loading duration (20 min). AAS concrete specimens exhibited greater creep at earlier loading ages and under higher stress levels. Among all specimens, 07G80TF00 exhibited the highest creep strain of 690 με. For low-stress specimens (0.3$f_{c}$ and 0.4$f_{c}$), creep coefficients were relatively small and showed minimal variation with loading age. As applied load increased, creep coefficients increased significantly. For specimens loaded to 0.5$f_{c}$ and 0.8$f_{c}$ at the same age, creep strain increased by at least 125 (e.g., 28G50TF10 vs. 28G80TF10). Loading age significantly affected creep response: under 0.8$f_{c}$ stress, specimens loaded at 7 d exhibited approximately 28% higher creep strain than those loaded at 28 d.

For specimens without fiber, average longitudinal creep coefficients at 7 d, 14 d, and 28 d were 0.36, 0.30, and 0.28, respectively. Increasing fiber content reduced creep coefficients. Specimens with 0.5% fiber exhibited average coefficients of 0.35, 0.28, and 0.26 at 7 d, 14 d, and 28 d, respectively. For 1.0% fiber content, coefficients were 0.35, 0.27, and 0.26. After 7 d, specimens with 0.5% and 1.0% fiber content showed 6.9% and 8.7% reductions in average creep coefficients, respectively, compared to non-fiber specimens.

The minimal variation in creep coefficients for control specimens under 0.3$f_{c}$ and 0.4$f_{c}$ suggests a nonlinear creep threshold of approximately 0.3$f_{c}$–0.4$f_{c}$ for AAS concrete, consistent with findings by [8,11,37]. The 28% higher creep strain in 7 d specimens compared to 28 d specimens under 0.8$f_{c}$ stress may be attributed to incomplete hydration of alkali-activated slag concrete at early ages, increasing susceptibility to damage under high stress.

Under sustained high stress, microcracks form and propagate during initial loading, evolving into macrocracks over time. This process induces creep damage and, for specimens loaded to 0.8$f_{c}$, leads to unconfined lateral expansion and eventual failure. Fiber reinforcement effectively suppresses lateral expansion, thereby slowing creep damage progression. At early loading ages (7 d), incomplete fiber-matrix integration resulted in creep coefficients comparable to non-fiber specimens. However, the significant reductions in creep coefficients after 7 d (6.9% for 0.5% fiber and 8.7% for 1.0% fiber) demonstrate that fibers constrain lateral expansion and improve long-term creep resistance under sustained high stress.

## 5. Influence of Fibers on Concrete Creep Damage

### 5.1. Mechanical Properties After Short-Term Sustained Loading

Damage mechanics is extensively employed to investigate the evolution of microdefects in materials and their impact on macroscopic mechanical properties. By introducing a damage variable as a physical quantity, this approach characterizes the evolution of microdefects. Based on Lemaitre’s strain equivalence principle, the effective stress $\bar{\sigma }$ in effective stress space can be utilized to analyze the mechanical behavior of damaged materials [39]. Under the strain equivalence hypothesis, replacing nominal stress in the constitutive model with effective stress transforms the constitutive relationship of damaged materials to that of undamaged materials. For concrete under uniaxial stress, the stress tensor and damage tensor reduce to scalar forms, and the isotropic elastoplastic damage expression is given by

(3) $$\sigma =\bar{\sigma }\left(1-d\right)=E_{0}\left(1-d\right)\left(\varepsilon -\varepsilon^{p}\right)=E_{0}\left(1-d\right)\varepsilon^{d}$$

where ε, $\varepsilon_{p}$, and $\varepsilon_{d}$ denote total strain, plastic strain, and damage-induced elastic strain, respectively; $E_{0}$ is the initial elastic modulus, *d* is the scalar damage variable, and $\bar{\varepsilon }$ is the equivalent strain, defined as

(4) $$\bar{\varepsilon }=\sqrt{\sum_{i=1}^{3}{\langle \varepsilon_{i}^{d}\rangle }^{2}}$$

where $\varepsilon_{i_{d}}$ represents the *i*-th damaged elastic principal strain related to Poisson’s ratio *v*, which can be calculated as follows:

(5) $$\bar{\varepsilon }=v\varepsilon^{d}$$

By evaluating the post-creep mechanical properties of concrete, the time-dependent damage mechanism can be indirectly observed, facilitating the characterization of specimen damage. After creep testing and unloading, specimens were reloaded to measure the peak stress and strain. To facilitate comparison across ages, normalized stress $\bar{\sigma }$ and strain $\bar{\varepsilon }$ were calculated using the same method as in Section 4.1. The elastic modulus *E* is another critical mechanical parameter. Normalized unloading ($E_{u}$) and reloading ($E_{r}$) moduli were calculated as

(6) $$\begin{array}{c} \bar{E_{u}}=\frac{E_{u}}{E_{m}}\\ \bar{E_{r}}=\frac{E_{r}}{E_{m}} \end{array}$$

where $E_{m}$ is the modulus during initial loading. The results are summarized in Table 8.

As shown in Table 8, low-stress specimens exhibit $\bar{\sigma }$ and $\bar{\varepsilon }$ values close to 1.0 with minimal age-dependent variation. Under medium stress (0.5$f_{c}$), specimens loaded at 7 d, 14 d, and 28 d show $\bar{\sigma }$ values of 0.98, 0.99, and 0.99 and $\bar{\varepsilon }$ values of 1.03, 1.03, and 1.02, respectively. At 0.7$f_{c}$, $\bar{\sigma }$ decreases and $\bar{\varepsilon }$ increases relative to medium-stress specimens. Specimens loaded at 0.8$f_{c}$ failed during the loading phase.

Low-stress specimens retain nearly intact modulus ($\bar{E_{u}}\approx 0.99$, $\bar{E_{r}}\approx 0.98$). At 0.5$f_{c}$, $\bar{E_{u}}$ and $\bar{E_{r}}$ are 0.96 and 0.87, respectively. For 0.7$f_{c}$, these values decrease to 0.88 and 0.69, with pronounced divergence between unloading and reloading moduli.

At 0.7$f_{c}$, specimens with 0.5% fiber content exhibit $\bar{\sigma }=0.82$ and $\bar{\varepsilon }=1.10$, representing 81% and 108% of control values, respectively. Specimens with 1.0% fiber content show $\bar{\sigma }=0.83$ and $\bar{\varepsilon }=1.09$. The elastic moduli for specimens with 0.5% fiber content are $\bar{E_{u}}=0.87$ and $\bar{E_{r}}=0.68$ (89% and 70% of control specimens), while specimens with 1.0% fiber content exhibit $\bar{E_{u}}=0.89$ and $\bar{E_{r}}=0.69$.

The near-unity $\bar{\sigma }$ and $\bar{\varepsilon }$ values under low stress indicate minimal damage accumulation, with modulus retention confirming structural integrity. The progressive reduction in $\bar{\sigma }$ and increase in $\bar{\varepsilon }$ at higher stress levels reflect accumulating damage, culminating in failure at 0.8$f_{c}$. These trends arise from competing mechanisms: hydration of gel materials around cracks and compression of capillary pores. Under medium stress, moisture facilitates crack closure through hydration and pore compression. However, under high stress, microcrack propagation outpaces healing, reducing both strength and stiffness. Gel water diffusion from interlayer spaces to capillary pores densifies the matrix under low stress but induces harmful stresses under high stress, accelerating microcrack formation.

Fiber reinforcement significantly mitigates damage under high stress. The elevated $\bar{\sigma }$ and reduced $\bar{\varepsilon }$ in fiber-reinforced specimens, coupled with higher modulus retention, demonstrate enhanced damage resistance. Fibers constrain microcrack propagation, promote hydration around cracks, and reduce stiffness degradation. The superior performance of 1.0% fiber specimens compared to 0.5% specimens confirms that increased fiber content improves the capacity to maintain mechanical properties after creep deformation, particularly under sustained high-stress conditions.

### 5.2. Poisson’s Ratio

The failure modes of AAS concrete specimens under monotonic loading and creep damage suggest that creep damage is primarily caused by the propagation of internal microcracks. Poisson’s ratio, defined as the negative ratio of transverse strain (perpendicular to the applied load) to axial strain (in the loading direction), remains nearly constant under low stress. However, under high stress, Poisson’s ratio increases considerably due to internal microcrack development. Thus, Poisson’s ratio can serve as an indirect indicator of microcrack progression in concrete. For cylindrical specimens, the nominal Poisson’s ratio is calculated as

(7) $$\varepsilon_{c}=\frac{\Delta C}{C}=\frac{2\pi \Delta R}{2\pi R}=\varepsilon_{r}$$

where *l* and Δl are the original length and its change, respectively; *C* and ΔC are the circumference and its change, respectively.

Since strain gauges were attached to the outer surface of the specimens, the measured strain corresponds to the strain at the outermost fibers, rather than the strain in the concrete core. When no macrocracks form, the circumferential strain is equivalent to the radial strain. However, when macrocracks develop, the circumferential and radial strains may differ, but their trends generally remain consistent. Therefore, the measured strain can still serve as an indirect indicator of macrocrack development during loading.

**Figure 7:**
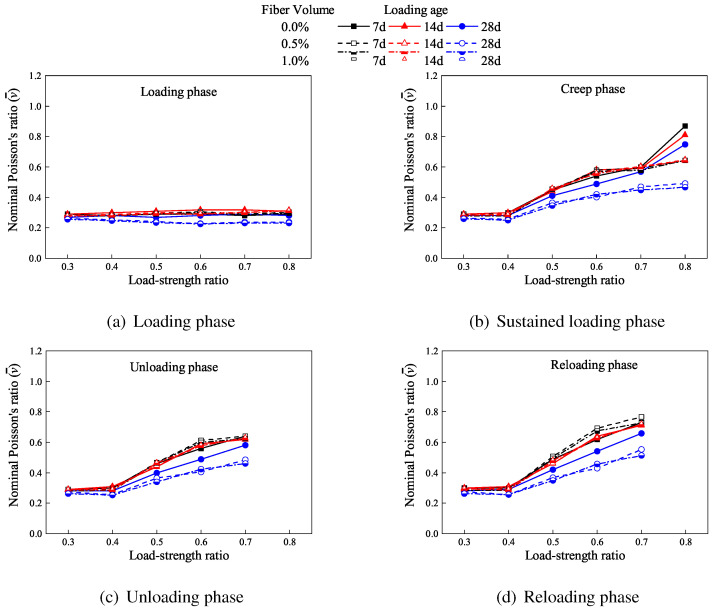
Nominal Poisson’s ratios for the specimens after different loading phases.

For normal-strength concrete, Poisson’s ratio begins to increase steadily when the applied stress exceeds a threshold stress level (referred to as the initial stress). At higher stress levels (critical stress), Poisson’s ratio exceeds 0.5, indicating volumetric expansion rather than continued contraction, which results from widespread microcrack propagation [24,40]. The nominal Poisson’s ratios of the specimens after the loading, holding, unloading, and reloading stages are shown in Figure 7.

As shown in Figure 7, the nominal Poisson’s ratio during the loading phase remains nearly constant across all specimens, with an average value of 0.28. For low-stress control specimens, the nominal Poisson’s ratio increases by 2% to an average of 0.29 during sustained loading, remaining stable during subsequent unloading (0.29) and reloading (0.30) phases. Medium-stress specimens (0.5$f_{c}$) exhibit progressive increases: average values reach 0.42 during sustained loading (49% increase from initial), 0.43 during unloading, and 0.45 during reloading. High-stress specimens (0.7$f_{c}$) show more pronounced increases, with averages of 0.56 during sustained loading (102% increase from initial), 0.59 during unloading, and 0.68 during reloading.

Fiber reinforcement reduces the nominal Poisson’s ratio across all phases. Compared to specimens without fiber, those with 0.5% fiber content exhibit reductions of 5%, 8%, and 18% during sustained loading at 7 d, 14 d, and 28 d ages, respectively, with similar reductions during unloading and reloading. Specimens with 1.0% fiber content show reductions of 6%, 11%, and 20% during sustained loading at the same ages, with comparable decreases in subsequent phases.

The near-constant nominal Poisson’s ratio (average 0.28) during the loading phase suggests minimal microcrack development at this stage [41]. The progressive increase in Poisson’s ratio for medium- and high-stress specimens during sustained loading, unloading, and reloading indicates ongoing damage accumulation in alkali-activated slag concrete even after load application. Notably, the initial stress threshold for significant damage in AAS concrete appears lower than that reported for conventional concrete. The substantial increases during unloading and reloading phases suggest continued microcrack propagation after load removal, likely driven by residual stresses concentrated near existing cracks. This phenomenon may explain the observed higher elastic modulus during unloading compared to loading.

Fiber reinforcement effectively constrains microcrack propagation, thereby limiting lateral expansion and reducing the nominal Poisson’s ratio. The dose-dependent reductions (greater with 1.0% vs. 0.5% fiber content) demonstrate that increased fiber dosage enhances crack restraint capacity. This results in smaller lateral expansion and lower Poisson’s ratios throughout all loading phases, confirming fibers’ role in mitigating damage progression in AAS concrete under sustained loading.

### 5.3. Non-Destructive Ultrasonic Test Results

Ultrasonic non-destructive testing has become a key method for assessing internal damage in concrete structures. The principle of ultrasonic testing relies on high-frequency mechanical waves emitted by the device, which reflect upon encountering interfaces. When concrete is highly compact, ultrasonic wave velocity remains relatively high. However, the presence of cracks or voids reduces wave velocity due to the increased path length and scattering. For creep-damaged specimens, internal defects (cracks and voids) become filled with air and water, further elongating the wave propagation path and reducing velocity. By normalizing ultrasonic wave velocities before and after creep tests and calculating the velocity variation rates, this study characterizes the levels of post-creep damage. The extra typical specimens were tested by ultrasonic device.

Table 9 compares the ultrasonic wave propagation time increments among the specimens. After short-term loading, all specimens show increased wave propagation times, indicating internal damage. High-stress specimens (07G70TF00, 07G70TF05, 07G70TF10, 28G70TF00, 28G70TF05 and 28G70TF10) display particularly pronounced effects: despite only 20 min of sustained loading, their propagation time increments exceed 0.5, indicating severe internal damage. Medium-stress specimens (07G50TF00, 07G50TF05 and 07G50TF10) show smaller increments; the average increase-ratio of the ultrasonic wave’s propagation time is 0.28, yet this value remains 177% of the control specimen (07G30CF00, 07G30CF05 and 07G30CF10), confirming significant damage even under moderate stress.

Early-age loading significantly influences creep damage. When comparing high-stress specimens loaded at 7 d and 28 d curing ages (07G70TF00 vs. 28G70TF00), the ultrasonic velocity ratio decreases with increasing loading age, with the 28 d specimen exhibiting 81% of the 7 d specimen’s ratio. Under high-stress loading, fiber-reinforced specimens demonstrate smaller ultrasonic propagation time increments compared to non-fiber specimens despite identical 20 min sustained loading durations. The average propagation time increment for fiber-reinforced specimens is 0.48, representing 89% of the value for specimens without fiber. Increasing fiber content further reduces propagation time increments: specimen 07G70TF10 shows a 6% reduction compared to specimen 07G70TF05.

The reduction in ultrasonic velocity ratio with increasing loading age reflects the rapid early-age strength development characteristic of alkali-activated slag concrete. Incomplete hydration during early stages produces higher initial compressive strength and elevated initial loads, though reduced long-term strength occurs due to higher activator requirements necessitated by slag’s poor workability [42,43]. As hydration progresses with age, initial defects diminish and propagation time increments during loading become more limited, resulting in less severe creep damage at later ages despite identical stress levels.

The reduced propagation time increments in fiber-reinforced specimens demonstrate fibers’ effectiveness in mitigating internal damage. Fibers inhibit microcrack propagation by delaying the transition from microcracks to macrocracks through crack-bridging mechanisms. The additional 6% reduction observed with increased fiber content (07G70TF10 vs. 07G70TF05) confirms that higher fiber dosage enhances microcrack suppression by redistributing internal stresses. These mechanisms align with established understanding of fiber reinforcement in cementitious materials, where fiber length and dosage critically influence crack control performance [44]. The findings collectively demonstrate that fiber incorporation substantially reduces internal damage evolution during sustained high-stress loading in alkali-activated slag concrete.

## 6. Conclusions

This study provides critical insights into the fundamental mechanisms governing creep damage evolution in fiber-reinforced alkali-activated slag concrete under sustained compressive loading. The following conclusions synthesize key findings to advance the theoretical understanding of time-dependent degradation in such materials

1. The stress-dependent response to short-term sustained loading reveals a damage threshold mechanism in alkali-activated matrices, where microcrack propagation transitions from beneficial densification at low stress levels (0.3$f_{c}$ to 0.4$f_{c}$) to destructive coalescence at higher intensities. This delineation establishes that creep damage is intrinsically governed by the competition between internal structure stabilization and fracture kinetics, offering a predictive framework for safe stress limits in structural design.
2. Fiber reinforcement functions as a microstructural regulator rather than a mere mechanical enhancer, effectively constraining microcrack growth and delaying macrocrack formation by redistributing localized stresses. However, the nonlinear attenuation of this protective effect with increasing fiber content underscores a critical optimization principle: maximal damage mitigation requires balancing fiber dosage against interfacial saturation thresholds to maintain crack-arresting efficiency.
3. Ultrasonic velocity degradation and Poisson’s ratio evolution collectively demonstrate that creep damage accumulation is predominantly driven by the loss of matrix cohesion under sustained loads, with fibers mitigating lateral expansion by preserving interfacial integrity. This mechanistic perspective redefines creep resistance in alkali-activated systems as a dynamic process of microcrack confinement, directly linking material composition to long-term dimensional stability and structural resilience.

This study examined limited fiber types and volume fractions under monotonic loading. Future research should prioritize long-term creep behavior under realistic service conditions, evaluate hybrid fiber systems for synergistic damage control, and establish predictive models linking microcrack evolution to macroscopic properties. Field validation through instrumented structural elements and investigation of interfacial transition zone modifications would further advance practical implementation of fiber-reinforced AAS concrete in sustainable infrastructure.

## Figures and Tables

**Table 1:** Material properties of the GGBFS, sodium silicate solution, and PP fibers [35].

Materials	Item	Unit (wt)	Value
GGBFS	SiO_2_	%	27.42
	Al_2_O_3_	%	18.44
	Fe_2_O_3_	%	0.34
	CaO	%	36.96
	MgO	%	8.74
	Na_2_O	%	0.61
	K_2_O	%	0.42
	SO_3_	%	2.48
	P_2_O_5_	%	0.02
	TiO_2_	%	0.74
	LOI	%	2.1
Sodium silicate solution	H_2_O	%	56.9
	Si_2_O	%	29.8
	Na_2_O	%	13.2
	Fe	%	0.02
	Baume	Baumé degree	50
	Density	g/${\mathrm{cm}}^{3}$	1.539
PP fiber	Length	mm	12
	Diameter	μm	60
	Density	g/${\mathrm{cm}}^{3}$	0.91
	Tensile strength	MPa	650
	Elastic modulus	MPa	4500

**Table 2:** Mix design of alkali activated slag concrete (kg/m^3^) [35].

Items	Mass
Fine aggregate	1110.65
GGBFS	666.39
Sodium hydroxide solution	93.29
Sodium silicate solution	479.80
Water	146.61
Water reducer	4.51
Water/binder	0.40

**Table 3:** Detailed summary of mid- or high-stress creep damage specimens.

Groups	ID	Age (Days)	Stress Ratio (%)	Fiber Volume (%)
Damage	07G50TF00	7	50	0
	07G50TF05			5
	07G50TF10			10
	07G60TF00		60	0
	07G60TF05			5
	07G60TF10			10
	07G70TF00		70	0
	07G70TF05			5
	07G70TF10			10
	07G80TF00		80	0
	07G80TF05			5
	07G80TF10			10
	14G50TF00	14	50	0
	14G50TF05			5
	14G50TF10			10
	14G60TF00		60	0
	14G60TF05			5
	14G60TF10			10
	14G70TF00		70	0
	14G70TF05			5
	14G70TF10			10
	14G80TF00		80	0
	14G80TF05			5
	14G80TF10			10
	28G50TF00	28	50	0
	28G50TF05			5
	28G50TF10			10
	28G60TF00		60	0
	28G60TF05			5
	28G60TF10			10
	28G70TF00		70	0
	28G70TF05			5
	28G70TF10			10
	28G80TF00		80	0
	28G80TF05			5
	28G80TF10			10

**Table 4:** Detailed summary of low-stress creep specimens.

Groups	ID	Age (Days)	Stress Ratio (%)	Fiber Volume (%)
Controlled	07G30CF00	7	30	0
	07G30CF05			5
	07G30CF10			10
	07G40CF00		40	0
	07G40CF05			5
	07G40CF10			10
	14G30CF00	14	30	0
	14G30CF05			5
	14G30CF10			10
	14G40CF00		40	0
	14G40CF05			5
	14G40CF10			10
	28G30CF00	28	30	0
	28G30CF05			5
	28G30CF10			10
	28G40CF00		40	0
	28G40CF05			5
	28G40CF10			10

**Table 5:** Peak strain and peak stress of monotonously loaded specimens. Values in parentheses represent the standard deviation of three specimens.

Age (Days)	Fiber Volume (%)	Peak Strain (με)	Peak Stress (MPa)	Elastic Modulus (GPa)
7	0.0	2715 (52)	51.8 (0.93)	24.1 (0.32)
	0.5	2762 (55)	55.4 (0.87)	25.2 (0.33)
	1.0	2776 (53)	57.5 (0.92)	26.3 (0.32)
14	0.0	2672 (29)	58.2 (0.92)	28.1 (0.28)
	0.5	2719 (29)	62.4 (0.87)	29.2 (0.25)
	1.0	2731 (27)	64.4 (0.88)	30.2 (0.32)
28	0.0	2692 (25)	65.2 (0.88)	31.7 (0.28)
	0.5	2664 (23)	70.2(0.85)	33.9 (0.22)
	1.0	2638 (23)	73.0 (0.86)	34.9 (0.24)

**Table 6:** The strain measurement results for the specimens. Values in parentheses represent the standard deviation of three specimens.

Specimen	Initial Strain *ε*_1_ (με)	Final Strain *ε*_2_ (με)	Irreversible Strain *ε_i_* (με)	Irreversible Ratio
07G30CF00	636 (12)	709 (14)	60 (3)	9.5%
07G30CF05	622 (11)	692 (13)	59 (3)	9.5%
07G30CF10	609 (10)	676 (12)	56 (3)	9.3%
07G40CF00	868 (15)	1006 (18)	85 (4)	9.8%
07G40CF05	846 (14)	980 (17)	81 (4)	9.5%
07G40CF10	829 (13)	958 (16)	79 (4)	9.5%
07G50TF00	1033 (18)	1254 (22)	129 (6)	12.5%
07G50TF05	1003 (17)	1216 (21)	119 (6)	11.9%
07G50TF10	975 (16)	1179 (20)	116 (6)	11.9%
07G60TF00	1240 (22)	1747 (30)	196 (9)	15.8%
07G60TF05	1212 (21)	1705 (29)	190 (9)	15.7%
07G60TF10	1185 (20)	1660 (28)	180 (8)	15.3%
07G70TF00	1381 (25)	1908 (33)	294 (13)	21.3%
07G70TF05	1349 (24)	1856 (32)	279 (12)	20.8%
07G70TF10	1318 (23)	1804 (31)	271 (12)	20.7%
14G30CF00	619 (12)	695 (14)	55 (3)	8.9%
14G30CF05	598 (11)	672 (13)	51 (3)	8.6%
14G30CF10	685 (13)	879 (16)	59 (3)	8.6%
14G40CF00	841 (14)	961 (17)	79 (4)	9.4%
14G40CF05	816 (13)	933 (16)	76 (4)	9.4%
14G40CF10	802 (13)	915 (15)	74 (4)	9.2%
14G50TF00	1208 (21)	1426 (25)	143 (7)	11.9%
14G50TF05	1172 (20)	1383 (24)	129 (6)	11.0%
14G50TF10	1158 (20)	1361 (24)	125 (6)	10.9%
14G60TF00	1307 (23)	1621 (28)	203 (9)	15.5%
14G60TF05	1272 (22)	1578 (27)	185 (8)	14.6%
14G60TF10	1250 (22)	1544 (26)	178 (8)	14.2%
14G70TF00	1408 (25)	1880 (32)	295 (13)	21.0%
14G70TF05	1373 (24)	1829 (31)	278 (12)	20.2%
14G70TF10	1346 (24)	1784 (30)	270 (12)	19.9%
28G30CF00	615 (12)	688 (14)	52 (3)	8.5%
28G30CF05	602 (11)	672 (13)	47 (3)	7.7%
28G30CF10	589 (11)	656 (12)	44 (3)	7.6%
28G40CF00	820 (14)	924 (16)	68 (4)	8.3%
28G40CF05	803 (13)	904 (16)	64 (4)	8.0%
28G40CF10	780 (13)	875 (15)	60 (4)	7.8%
28G50TF00	1074 (19)	1325 (23)	111 (5)	10.3%
28G50TF05	1042 (18)	1283 (22)	107 (5)	10.3%
28G50TF10	1019 (18)	1248 (22)	102 (5)	10.1%
28G60TF00	1304 (23)	1604 (28)	182 (8)	13.9%
28G60TF05	1273 (22)	1563 (27)	167 (8)	13.1%
28G60TF10	1237 (22)	1510 (26)	160 (8)	12.9%
28G70TF00	1379 (25)	1734 (30)	254 (11)	18.4%
28G70TF05	1350 (24)	1690 (29)	247 (11)	18.3%
28G70TF10	1316 (24)	1638 (28)	237 (10)	17.9%

The specimen loaded with 0.8$f_{c}$ failed during the sustained loading process.

**Table 7:** Creep strain and creep coefficient of specimens. Values in parentheses represent the standard deviation of three specimens.

Specimen	Creep Strain (με)	Creep Coefficient	Specimen	Creep Strain (με)	Creep Coefficient	Specimen	Creep Strain (με)	Creep Coefficient
07G30CF00	73 (4)	0.11	14G30CF00	76 (4)	0.12	28G30CF00	73 (4)	0.12
07G30CF05	70 (4)	0.11	14G30CF05	74 (4)	0.12	28G30CF05	70 (4)	0.12
07G30CF10	67 (4)	0.11	14G30CF10	94 (5)	0.12	28G30CF10	67 (4)	0.11
07G40CF00	138 (7)	0.16	14G40CF00	120 (6)	0.14	28G40CF00	104 (5)	0.13
07G40CF05	134 (7)	0.16	14G40CF05	117 (6)	0.14	28G40CF05	101 (5)	0.13
07G40CF10	129 (7)	0.16	14G40CF10	113 (6)	0.14	28G40CF10	95 (5)	0.12
07G50TF00	221 (11)	0.21	14G50TF00	218 (11)	0.18	28G50TF00	251 (12)	0.23
07G50TF05	213 (11)	0.21	14G50TF05	211 (11)	0.18	28G50TF05	241 (12)	0.23
07G50TF10	204 (10)	0.21	14G50TF10	203 (10)	0.18	28G50TF10	229 (11)	0.23
07G60TF00	507 (25)	0.41	14G60TF00	314 (16)	0.24	28G60TF00	300 (15)	0.23
07G60TF05	493 (25)	0.41	14G60TF05	306 (15)	0.24	28G60TF05	290 (15)	0.23
07G60TF10	475 (24)	0.40	14G60TF10	294 (15)	0.24	28G60TF10	273 (14)	0.22
07G70TF00	527 (26)	0.38	14G70TF00	472 (24)	0.34	28G70TF00	355 (18)	0.26
07G70TF05	507 (25)	0.38	14G70TF05	456 (23)	0.33	28G70TF05	340 (17)	0.25
07G70TF10	485 (24)	0.37	14G70TF10	438 (22)	0.32	28G70TF10	322 (16)	0.25
07G80TF00	690 (35)	0.43	14G80TF00	662 (33)	0.38	28G80TF00	572 (29)	0.35
07G80TF05	668 (33)	0.42	14G80TF05	643 (32)	0.38	28G80TF05	551 (28)	0.34
07G80TF10	641 (32)	0.41	14G80TF10	615 (31)	0.37	28G80TF10	517 (26)	0.33

**Table 8:** The detailed normalized mechanical properties after sustained loading.

Specimen	Normlized Data			
	Peak Strain $\bar{\varepsilon_{0}}$	Peak Strength $\bar{\sigma_{0}}$	Unloading Elastic Modulus $\bar{Eu}$	Reloading Elastic Modulus $\bar{Er}$
07G30CF00	1.02	1.04	1.04	1.00
07G30CF05	1.02	1.02	1.01	0.98
07G30CF10	1.02	1.04	0.99	0.96
07G40CF00	1.03	1.00	1.03	1.00
07G40CF05	1.03	1.00	1.00	0.98
07G40CF10	1.03	1.01	0.98	0.97
07G50TF00	1.05	0.94	0.99	0.89
07G50TF05	1.05	0.96	0.96	0.87
07G50TF10	1.04	0.98	0.93	0.85
07G60TF00	1.08	0.89	0.97	0.78
07G60TF05	1.07	0.90	0.95	0.77
07G60TF10	1.07	0.91	0.93	0.76
07G70TF00	1.12	0.86	0.93	0.72
07G70TF05	1.11	0.81	0.91	0.70
07G70TF10	1.10	0.83	0.88	0.69
14G30CF00	1.01	0.99	1.01	1.00
14G30CF05	1.01	1.03	0.99	0.97
14G30CF10	1.04	1.02	1.01	1.00
14G40CF00	1.02	0.99	1.01	1.00
14G40CF05	1.02	0.99	0.98	0.98
14G40CF10	1.02	0.99	0.96	0.97
14G50TF00	1.05	1.01	0.98	0.88
14G50TF05	1.04	0.97	0.95	0.86
14G50TF10	1.04	1.00	0.94	0.85
14G60TF00	1.07	0.89	0.96	0.78
14G60TF05	1.07	0.91	0.93	0.76
14G60TF10	1.06	0.88	0.91	0.75
14G70TF00	1.12	0.85	0.91	0.71
14G70TF05	1.10	0.82	0.89	0.70
14G70TF10	1.10	0.84	0.88	0.69
28G30CF00	1.00	1.01	1.00	0.99
28G30CF05	1.00	1.04	0.97	0.96
28G30CF10	0.99	1.00	0.95	0.94
28G40CF00	1.00	1.00	1.00	0.99
28G40CF05	1.00	1.01	0.98	0.98
28G40CF10	1.00	1.03	0.95	0.95
28G50TF00	1.02	0.98	0.98	0.88
28G50TF05	1.02	1.01	0.96	0.87
28G50TF10	1.02	0.97	0.93	0.85
28G60TF00	1.05	0.93	0.93	0.78
28G60TF05	1.05	0.92	0.91	0.76
28G60TF10	1.05	0.91	0.88	0.74
28G70TF00	1.08	0.87	0.89	0.69
28G70TF05	1.08	0.84	0.87	0.68
28G70TF10	1.07	0.82	0.85	0.66

The specimen loaded with 0.8$f_{c}$ failed during the sustained loading process.

**Table 9:** Comparison of increase-ratio of ultrasonic wave’s propagation time for specimen.

Specimen		07G30CF00	07G30CF05	07G30CF10	07G50TF00	07G50TF05	07G50TF10
Point	1	0.17	0.17	0.13	0.31	0.28	0.31
	2	0.16	0.18	0.13	0.26	0.28	0.26
	3	0.18	0.16	0.17	0.27	0.28	0.29
	4	0.16	0.18	0.14	0.30	0.25	0.27
	5	0.15	0.18	0.16	0.30	0.28	0.30
	6	0.22	0.18	0.14	0.29	0.31	0.27
	7	0.17	0.16	0.15	0.30	0.28	0.25
	8	0.15	0.12	0.17	0.28	0.28	0.23
	9	0.14	0.13	0.11	0.35	0.30	0.22
Mean value		0.16	0.16	0.15	0.30	0.28	0.27
**Specimen**		**07G70TF00**	**07G70TF05**	**07G70TF10**	**28G70TF00**	**28G70TF05**	**28G70TF10**
Point	1	0.61	0.78	0.68	0.42	0.49	0.32
	2	0.70	0.72	0.54	0.43	0.51	0.44
	3	0.64	0.77	0.54	0.48	0.46	0.60
	4	0.70	0.55	0.66	0.49	0.56	0.47
	5	0.72	0.71	0.62	0.50	0.51	0.42
	6	0.62	0.57	0.65	0.54	0.54	0.52
	7	0.62	0.79	0.70	0.60	0.52	0.42
	8	0.69	0.63	0.61	0.70	0.54	0.35
	9	0.60	0.23	0.61	0.70	0.39	0.69
Mean value		0.66	0.64	0.62	0.54	0.50	0.47

## Data Availability

The original contributions presented in this study are included in the article. Further inquiries can be directed to the corresponding author.
